# Depth perception of stereoscopic transparent stimuli with frame manipulation

**DOI:** 10.1038/s41598-024-57283-9

**Published:** 2024-03-20

**Authors:** Saori Aida, Shunta Fukamachi, Koichi Shimono

**Affiliations:** 1 Graduate School of Sciences and Technology for Innovation, Yamaguchi University, 2-16-1 Tokiwadai, Ube, 753-8611 Japan; 2 Graduate School of Marine Science and Technology, Tokyo University of Marine Science and Technology, 2 Etchujima, Koto-ku, Tokyo, 135-8533 Japan

**Keywords:** Psychology, Human behaviour

## Abstract

Depth perception is crucial in human vision, allowing us to move and interact with our 3-D surroundings. We used a stereoscopic transparent stimulus comprising parallel overlapping transparent stereoscopic surfaces (POTS) to understand depth perception better. The study focused on exploring the effect of a surrounding frame on the perceived depth of a POTS configuration. The research was based on a proposed idea that explains an "off-frame" effect: a frame at a different depth from a 2-D photograph depicting a 3-D scene increases its apparent depth qualitatively. The idea assumes that processing the disparity between a frame and a photo reduces the reliability of the photograph's flatness cues and increases depth magnitude in depth cue integration. We examined whether the idea can be applied to a 3-D POTS with the flatness cue as the constant accommodation. Through three experiments, the study showed that frames impact the perceived depth magnitude of a POTS configuration. More specifically, the depth magnitude increases as the frame's disparity concerning the monitor plane increases and decreases as the frame's size increases. We discussed the results in the context of depth cue combination.

## Introduction

Wheatstone^1^ demonstrated that a pair of stimuli can produce a vivid three-dimensional (3-D) impression by generating retinal images slightly different between the two eyes, equivalent to those produced by objects with depth. Although Wheatstone’s first stereograms contained monocular contours that could provide monocular pictorial depth cues, Julesz^2^ developed the stereogram consisting of random dots that excluded the monocular cues and showed that there exists a purely binocular cue to mediate the 3-D impression. This cue has been known as binocular (horizontal) disparity that leads to depth perception or binocular stereopsis.

The depth magnitude can be calculated geometrically from a binocular disparity between objects, absolute (egocentric) distance to them, and the interpupillary distance. The visual system utilizes the disparity to perceive depth quantitatively when its amount is small^3–12^. The magnitude of perceived depth deviates from the geometric prediction for some stimulus properties^6,11,13,14^, and under some viewing conditions^15–19^. This deviation is often attributed to the availability of egocentric distance cues^15,16^ and their conflict^10^, participants’ experience^10,17^, and tasks^10,16^. The deviation is interpreted as indicating that the visual system utilizes a perceived egocentric distance and/or a perceived disparity to calculate the apparent depth magnitude^15,20–22^ and helps us to understand the mechanism(s) to process the disparity^3–12^.

As one of the stereoscopic stimuli showing the discrepancies, Aida et al.^21,22^ identified a POTS configuration, which consists of random dots and depicts parallel overlapping, transparent stereoscopic surfaces. When fused, a participant perceives multiple overlapping surfaces with depth within the same region of the visual field^21–27^. Even when the disparity between the closest and the farthest surfaces of a POTS remains constant, the depth magnitude between them decreases as the surface number of the POTS increases. This reduction in depth does not align with the geometric prediction.

We examined whether and how a frame impacts the perceived depth of a POTS. When a 2-D stimulus (a picture) depicting a 3-D scene is surrounded by a frame, the frame can increase the apparent depth impression of the scene^28^. This “off-frame” effect was explained in the framework of the depth-cue combination, which assumes the frame’s disparity cue, the photo’s pictorial depth cues, and the photo’s flatness cues (such as the photo’s constant accommodation and zero disparity) are combined depending on their reliability at the stage of cue combination; the disparity between a frame and a framed stimulus decreases the reliability of the flatness information of the framed, increases the reliability of photo’s depth cue information, and enhances its depth impression. In this assumption, a frame and a 2-D stimulus are at different places to each other. Note that the combination is assumed to operate for the different depth cues in the same scene in the depth cue combination model^29–31^.

We applied the reliability assumption to the depth perception of a POTS. Compared with binocular viewing in everyday 3-D space, stereoscopic viewing is known to create vergence-accommodation mismatch^32^, where two types of flatness information can be produced, that is, no variation in blur in retinal images and no correlation between the depth information from vergence and accommodation^31^. If a frame decreases the reliability of the flatness information of a POTS and increases the reliability of its depth information, the perceived depth magnitude of the POTS would increase as the off-frame effect of a picture. We used a POTS because we expected that the effect, if any, could help us understand the mechanism(s) mediating its depth reduction phenomenon.

We also examined how the size of a frame influences the perceived depth of a POTS. While many studies have examined how a slanted frame affects the depth magnitude of a framed stimulus^3,33^, very few have explored how a frame on a front plane affects the perceived depth of a stimulus. In one of the few studies, Nate^34^ found that the size of a frame in a front surface affected the perceived depth magnitude of a 3-D target. However, it is not yet known whether that result was due solely to the frame size, as Nate^34^ also manipulated the size of the stimulus (target and frame). If the size of a frame impacts the apparent depth, it could help us better understand the depth enhancement effect of a frame.

This study investigated whether a frame surrounding a POTS configuration can affect the inside configuration's perceived depth and how the frame's position and size can impact the perceived depth. The frame was positioned either in front of, behind the POTS, or on a front-parallel plane with zero disparity, dividing the POTS depth-wise. In Experiment 1, participants reproduced the perceived depth magnitude of framed and frameless POTS under four frame conditions. Experiment 2 measured the total disparity of a framed two- or three-POTS that produced the same apparent depth as a frameless POTS under the front and equidistant frame conditions. Experiment 3 measured the depth of framed and frameless two- or three-POTS configurations as in Experiment 1. We used three distinct-sized frames under the equidistant frame condition.

## Results

In each experiment, we investigated if participants accurately identified the surface count for different POTS configurations initially. All participants provided the correct number of surfaces, indicating their skilled observation of stereo surfaces.

### Experiment 1

Participants reproduced the depth magnitude of the POTS stimulus. Experiment 1 used the perceived depth magnitude averaged for each condition and participant as the basic unit of analysis. There were three conditions: each with four frame positions (frameless, front, back, and equidistant), three POTS configurations (two-, three-, and four-), and three total disparities (small, medium, and large). We conducted a three-way repeated measures analysis of variance 4 (frame) × 3 (surface) × 3 (disparity) on the basic unit. We found that the main effects of frame [*F* (3, 24) = 17.41, *p* < 0.01, *η*^*2*^ = 0.08], surface [*F* (2, 16) = 17.66, *p* < 0.01, *η*^*2*^ = 0.04], and disparity [*F* (2, 16) = 31.15, *p* < 0.01, *η*^*2*^ = 0.26] were statistically significant. Multiple comparisons using Ryan's method showed that 1) the difference in the perceived depth between any two frame conditions was statistically significant for all comparisons (*p* < 0.05) except between the front and back frame conditions, 2) the difference between any two POTS conditions was statistically significant for all comparisons (*p* < 0.05), and 3) the difference between two total disparity conditions was statistically significant for all comparisons (*p* < 0.05).

**Figure 1:**
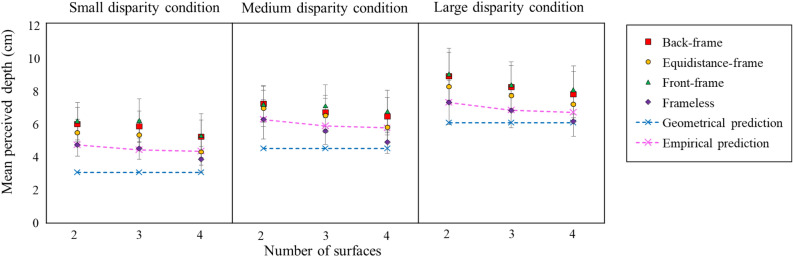
The mean perceived depths as a function of the number of surfaces of a POTS configuration for the small, medium, and large disparity conditions. The purple rhombus, green triangle, orange circle, and red square show the results of the frameless, front-frame, equidistant-frame, and back-frame conditions, respectively. Each of those symbols represents the mean value across participants. Error bars signify the 95% confidence interval. The blue and pink cross marks indicate the geometrical prediction and the prediction from empirical data, respectively. We described the calculation method in the “Results” section. Dotted lines connected the cross marks with the same color for the descriptive purpose.

Figure 1 shows the mean perceived depth across participants as a function of the POTS’ surface number separately for four frame conditions for each of the three disparity conditions. Figure 1 also shows the predictions from the geometry and empirical data. The geometrical prediction was made using the ordinary standard equation, assuming the interpupillary distance was 6.4 cm. The empirical prediction was made using the results of Aida et al.’s^22^ Experiment 4, where each POTS had no frame. First, we calculated the ratio of the perceived depth magnitude of the two-POTS to that of the two-, three-, or four-POTS. The magnitude was the average of the two disparity conditions. Then, we multiplied each of the three ratios by the mean magnitude of the frameless two-POTS in this experiment. Note that the values of the empirical prediction were the same at each disparity condition.

The figure indicates that (1) as the prediction from empirical data, the perceived depth decreases with the POTS' surface number, (2) the perceived depth is larger for the framed than the frameless, (3) the perceived depth for the frameless is larger than the geometrical prediction and similar to the empirical prediction, (4) the perceived depth of the front and back frames is similar to each other and larger than that of the equidistant-frame, and 5) the mean perceived depth increased as the total disparity increased.

We further computed the slopes of the regression lines using the data from the three surface conditions shown in Fig. 1. We performed a two-way repeated measures analysis of variance 4 (frame) × 3 (disparity) on the slopes. We found no significant main effects of frame [*F* (3, 24) = 1.17, *p* = 0.34, *η*^*2*^ = 0.03], disparity [*F* (2, 16) = 0.26, *p* = 0.77, *η*^*2*^ < 0.01], and its interaction [*F* (6, 48 = 1.82, *p* = 0.11, *η*^*2*^ = 0.03]. The non-significances suggest that the degree of the frame’s effect on the POTS’ depth reduction phenomenon effect was constant and did not reflect on the slope of the regression lines.

### Experiment 2

Participants compared side-by-side framed and unframed POTS and reported which appeared deeper. The disparity of the framed POTS was incrementally adjusted to derive the point of subjective equality (PSE). The PSE was calculated from a psychometric function of the percentage of responses where the framed POTS' depth was greater than the frameless POTS' depth against the disparity of the frameless POTS. The psychometric function fitted was sigmoid. The disparity that produced a 50% response in the function was the PSE. We subtracted the PSE of the framed POTS from the frameless POTS disparity for each condition and participant. We used the difference to validate the depth enhancement effect observed in Experiment 1. The Experiment had three conditions: each with two frame positions (front (26.73 arcmin) and equidistant (0.00 arcmin)), two surface configurations (two- and three-POTS), and three total disparities (small, medium, and large).

We conducted a three-way repeated measures analysis of variance 2 (frame) × 2 (surface) × 3 (disparity) on the subtracted value or a bias of PSE. We found that the main effects of frame [*F* (1, 8) = 5.33, *p* < 0.05, *η*^*2*^ = 0.11], but not of surface [*F* (1, 8) = 3.16, *p* = 0.11, *η*^*2*^ = 0.03] and disparity [*F* (2, 16) = 1.63, *p* = 0.23, *η*^*2*^ = 0.02]. There was a statistically significant interaction between the frame and surface [*F* (1, 8) = 19.68, *p* < 0.01, *η*^*2*^ = 0.04]. A simple main effect showed a significant difference between the two and three surfaces at the equidistant-frame condition [*F* (1, 8) = 14.11, *p* < 0.01, *η*^*2*^ = 0.14] but not at the front-frame condition [*F* (1, 8) = 0.01, *p* = 0.94, *η*^*2*^ < 0.01]. It also showed a significant difference between the two frame conditions at the three-POTS configuration [*F* (1, 8) = 16.52, *p* < 0.01, *η*^*2*^ = 0.34] but not at the two-POTS configuration [*F* (1, 8) = 0.70, *p* = 0.43, *η*^*2*^ = 0.02].

**Figure 2:**
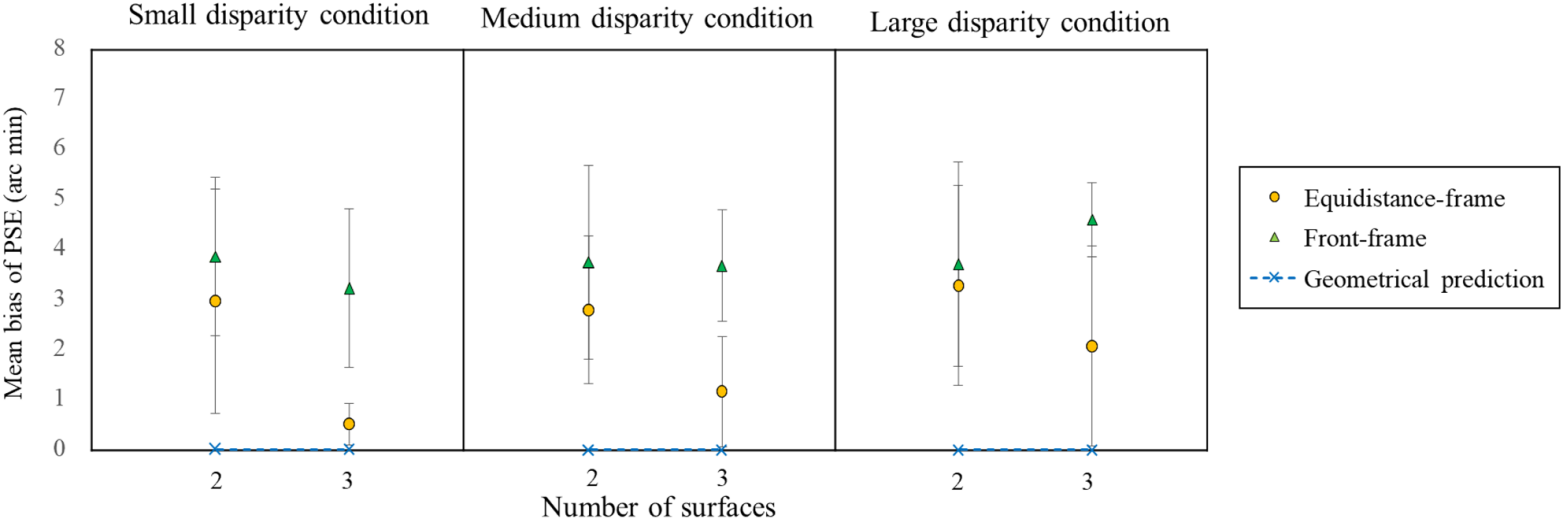
The mean biases of PSEs of two-POTS and three-POTS configurations as a function of two frame conditions for the small, medium, and large disparity conditions. The blue triangle and orange circle show the results of the front- and equidistance-frame conditions, respectively. Error bars signify the 95% confidence interval. The blue cross, connected dotted line, indicates the geometrical prediction as in Fig. 1.

Figure 2 shows the mean bias of the PSE over participants as a function of the surface number of the POTS configuration for each of the three disparity conditions. Figure 2 also shows the predictions from the geometry; when the depth, viewing distance, and interpupillary distance are the same, the disparity should be the same between the framed and frameless POTS, and the difference between the two would be zero. The figure indicates that (1) the mean bias was larger than the geometrical prediction, (2) the difference in the mean bias between the two- and three-POTS was more noticeable for the equivalent-frame than the front-frame, and (3) the difference in mean bias between the equivalent-frame and front-frame was more prominent for three-POTS than two-POTS.

We assessed the 95% CI of the mean bias of PSE for each condition. As evidenced in Fig. 2, the lower value of the CI was more than zero for all viewing conditions. The result indicates that it needed a smaller disparity than the frameless POTS for the framed POTS to be perceived to be the same depth as the frameless, that is, a frame surrounding a frame increased the perceived depth magnitude of a POTS inside. This result is consistent with the results of Experiment 1 on the effect of frame depth enhancement.

### Experiment 3

Participants reproduced the depth magnitude of the POTS stimulus. Experiment 3 used the perceived depth magnitude averaged for each condition and participant as the basic unit of analysis. There were three conditions: each with four frames (frameless, small, medium, and large), two surface configurations (two- and three-POTS), and three total disparities (small, medium, and large). A three-way repeated measures analysis of variance 4 (frame × 2 surface × 3 disparity) on the basic score revealed that (1) the main effects of frame [*F* (3, 24) = 13.80, *p* < 0.01, *η*^*2*^ = 0.03], surface [*F* (1, 8) = 28.67, *p* < 0.01, *η*^*2*^ = 0.03], disparity [*F* (2, 16) = 35.43, *p* < 0.01, *η*^*2*^ = 0.28], and (2) interaction between frame and disparity [*F* (6, 48) = 2.78, *p* < 0.05, *η*^*2*^ < 0.01] were statistically significant. Simple main effect analysis revealed that the four frame conditions were significantly different for the small [*F* (3, 24) = 4.59, *p* < 0.05, *η*^*2*^ = 0.03], medium [*F* (3, 24) = 10.27, *p* < 0.01, *η*^*2*^ = 0.02], and large disparity [*F* (3, 24) = 15.04, *p* < 0.01, *η*^*2*^ = 0.05]. The three disparity conditions were also statistically significantly different for the frameless [*F* (2, 16) = 29.25, *p* < 0.01, *η*^*2*^ = 0.23], small-frame [*F* (2, 16) = 35.49, *p* < 0.01, *η*^*2*^ = 0.31], medium-frame [*F* (2, 16) = 32.50, *p* < 0.01, *η*^*2*^ = 0.31], and large-frame [*F* (2,16) = 29.70, *p* < 0.01, *η*^*2*^ = 0.29]. Multiple comparisons using Ryan's method showed that: (1) there were significant differences between the small-frame and frameless and between the medium-frame and frameless for each size condition, (2) between the small- and large-frames and between the medium- and large-frames for each size condition except for between the small and large-frames for the medium size condition, and (3) between the frameless and large-frame for the medium and large size conditions (*p* < 0.05). The multiple comparisons also showed that the difference in the perceived depth between any two surface conditions and any two disparity conditions was statistically significantly different for all comparisons (*p* < 0.05).

**Figure 3:**
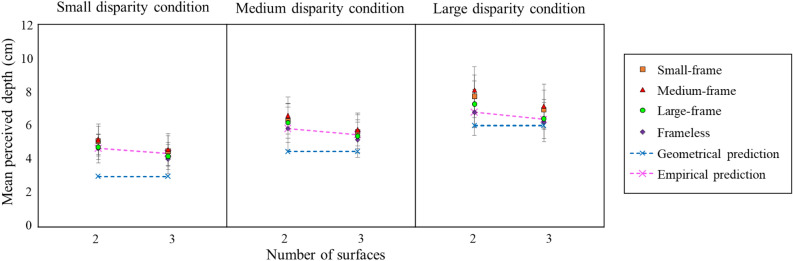
The mean perceived depths as a function of the number of surfaces of a POTS configuration for the small, medium, and large disparity conditions. The blue rhombus, green circle, red triangle, and orange square show the results of the frameless, large-frame, medium-frame, and small-frame conditions, respectively. Error bars signify the 95% confidence interval. The blue and pink cross marks indicate the geometrical prediction and the prediction from empirical data, respectively, as in Fig. 1.

Figure 3 shows the mean perceived depth across participants as a function of the POTS' surface number separately for four frame conditions for each of the three disparity conditions. Figure 3 also shows the predictions from the geometry and Aida et al.’s^22^ empirical data. The geometrical prediction was made as in Fig. 1. The empirical prediction was also computed as in Fig. 1 except that we calculated the ratio to the two-POTS and three-POTS. The figure revealed that (1) as the prediction from empirical data, the mean perceived depth is greater for the two-POTS configuration than the three-POTS configuration in all conditions, (2) the perceived depth for the frameless is larger than the geometrical prediction and similar to the empirical prediction, (3) the perceived depths of the small-frame and medium-frame are greater than those of the frameless and large-frame conditions in general, and (4) the perceived depth increased as the total disparity increased for all frame and surface conditions.

We calculated the slopes of the lines connecting two data points for the two surface conditions shown in Fig. 3. We performed a two-way repeated measures analysis of variance 4 (frame) × 3 (disparity) on the slopes. We found no significant main effects of frame [*F* (3, 24) = 0.49, *p* = 0.69, *η*^*2*^ = 0.01], disparity [*F* (2, 16) = 2.97, *p* = 0.08, *η*^*2*^ = 0.03], and its interaction [*F* (6, 48 = 1.05, *p* = 0.41, *η*^*2*^ = 0.02]. The non-significances in this experiment are consistent with those in Experiment 1.

## Discussion

We studied the effect of a frame on the perceived depth magnitude of a stereoscopic stimulus in three experiments. The stimulus consisted of random dots, depicted parallel overlapping, transparent stereoscopic surfaces (POTS), and was presented with or without a frame. We found that (1) the framed stimulus had a larger depth magnitude than the frameless stimulus (Experiments 1, 2, and 3); (2) the depth magnitude for a front or back frame is similar to each other and more extensive than that with a frame at a display plane (Experiment 1); (3) the enhancement of the depth magnitude for a framed disappeared when the frame’s size was relatively large (Experiment 3), and (4) the apparent depth magnitude of the POTS decreased as the number of surfaces increased (see Figs. 1,3).

The first and second findings show that a POTS configuration enclosed within a frame leads to an increased perception of depth magnitude, as predicted by the reliability assumption of depth cue integration in a scene. The reliability explains the off-frame effect whereby the perceived depth of contents in a 2-D photo is heightened when presented with a frame in front or behind the photo. Conversely, the effect is less eminent when the frame is on the same depth plane as the photo. The findings align with those in Shimono et al.^28^ and suggest that when a stimulus contains both flatness and depth information, and a surrounding frame contains disparity information, the reliability of the flatness information while the depth information decreases. As a result, the perceived depth of the stimulus increases qualitatively or quantitatively. This notion implies that the perceived depth magnitude of various stimuli that contain flatness information can be enhanced by inducing a surrounding frame. Future studies can use a stereoscopic, motion parallax, or 2D stimulus containing a pictorial depth cue to examine the reliability assumption.

One may, however, wonder whether the “total size” of the stimulus (POTS and frame) affected the depth enhancement effect of a frame. If the total size affected the perceived depth, the findings could be due to the size difference but not the frame surrounding a POTS. In the control experiment, we asked seven participants to compare the apparent depth magnitude of two different-sized two-POTS configurations (small and large) presented side-by-side. The sizes of the small and large POTS were the same as those of the POTS without and with a frame used in Experiment 2, respectively, and the disparity of the large POTS was constant (18.34'), and that of the small POTS varied from 6.11 to 30.60 in five steps. The disparity of the small POTS and its location were randomly selected from five disparities and two locations (right and left), respectively, with five repetitions. The result showed that the mean disparity of the small POTS was 18.34 (95% CI, 18.15–18.53), which was the same value as the large POTS, indicating that the total size of the POTS did not affect the perceived depth magnitude.

In Experiments 1 and 3, we did not control the eye positions, and it is essential to consider their potential influence on a POTS configuration's perceived depth. However, in Experiment 2, we observed the depth enhancement effect even when we presented the framed and frameless POTS side-by-side. Aida et al.^22^ also reported the depth reduction phenomenon when two- and three-POTS or three- and four-POTS were presented side-by-side. As both POTS would have been similarly affected by the vergence eye movements, if any, it is unlikely that this impacted their apparent depth differently.

We conducted two control experiments using a nonius stimulus to support our argument. The nonius stimulus has often been used to monitor binocular eye position subjectively^35,38^, although it has certain limitations^39–42^. The stimulus consisted of a binocular horizontal bar (0.35 × 0.05 arcdeg) with a monocular vertical line (0.05 × 0.18 arcdeg) above or below at its center. The stimulus was surrounded by a binocular square (0.35 × 0.35 arcdeg, 0.05 arcdeg stroke width), which facilitates binocular fusion and helps lock the vergence position^35–37^. We presented two POTS with the nonius and square stimuli between the POTS. In the first and second experiments, we used the frameless and framed two-POTS and the frameless two- and three-POTS, respectively. The first and second experiments examined whether the different eye positions affected the depth enhancement effect and depth reduction phenomenon, respectively. The nonius and the square were presented at the same depth plane as the front frame, the monitor plane, and the back frame in the first and as the front surface, the monitor plane, and the back surface in the second. The sizes of the POTS and frame, the task, and the experimental procedure were essentially the same as in Experiment 2. At the same time, we asked eight participants to respond when the nonius lines appeared aligned, assuming that the binocular eye position was the desired position when they appeared aligned.

The first control showed that the mean disparity of the frameless producing the same apparent depth as the framed two-POTS with the 18.34 arcmin disparity was 21.97 arcmin (95% CI, 20.02–23.92), 21.48 arcmin (95% CI, 18.97–23.99), or 21.48 arcmin (95% CI, 20.11–22.85) for the front, monitor-plane, or back vergence position, respectively. The vergence eye position did not affect the depth enhancement effect. The second control indicated that the mean disparity of the two-POTS producing the same apparent depth as the three-POTS with the 18.34 arcmin disparity was 17.16 arcmin (95% CI, 16.03–18.29), 16.63 arcmin (95% CI, 15.27–18.00), or 15.72 arcmin (95% CI, 14.67–16.77) for the front, monitor-plane, or back vergence position, respectively. The depth reduction phenomenon was observed when the vergence was kept constant.

The analysis of the slopes of the regression lines in Experiment 1 indicates that the slope is constant among the four frame conditions. This constant slope suggests that the disparity processing for a surrounding frame is independent of that for a POTS inside and combines at the stage of depth cue integration. The third finding suggests that the area where the combining process covers is limited; the frame may contribute less to the perceived depth of the POTS as the separation between the frame and the POTS as shown in Fig. 3.

Our frame effect is similar to depth contrast in which an inclined frame affects the depth perception of a framed stimulus^3,43–45^. For example, Werner^43^ demonstrated that when a slanted frame was presented around a horizontal line with zero disparity, it appeared slanted in depth in the opposite direction. The slanted frame is normalized as a new reference as a front-parallel plane^3,27–29^; when a slanted frame becomes a new norm as a reference, the line will slant. We can apply the normalization to the depth enhancement effect. In that case, we can use the idea that depth perception is described geometrically in terms of perceptual variables^46^, which assumes that if the same physical egocentric distance to a stimulus is the same, the perceived egocentric distance can change and scale the disparity information. Thus, if the frame’s position affects the apparent egocentric distance to a POTS configuration, the perceived magnitude of the depth of a POTS configuration can differ. The new "reference" would be the perceived distance if this is the case.

The fourth finding agrees well with the previous finding that the depth magnitude of a POTS configuration reduces as the number of its surfaces increases from two to four^21,22^; the degree of reduction predicted from the previous finding was similar to the present results as shown in Figs. 1,3. One may think that the depth reduction can be due to incorrect matches (often called ghost images or false projection), which are assumed to obstruct the visual system from finding correct matches; an increase in dot density could deteriorate stereopsis^23,24^. In the present study, however, we kept the number of elements in each eye constant; the total density of elements of the 2-, 3-, and 4-POTS configurations and the total number of correct matching and incorrect matching dots were the same among the three POTS. Furthermore, the dot density of the surface was reported not to affect the reduction of the perceived depth in the three-POTS configuration^22^. Consequently, the depth reduction phenomenon may not be due to the false projection.

In this study, reducing the surface number of a POTS decreased the element number (or dot density) per surface, which could have affected the depth reduction phenomenon. Aida et al.^22^, however, found no effect of the dot density with the depth reproduction method; each of the three-POTS with different dot densities (0.37, 0.73, or 1.1 dots/deg^2^) appeared at the same depths and shallower than the two-POTS with a dot density (1.1 dots/deg^2^) when they had the same disparity. We confirmed their finding with the depth-discrimination method using a dot density close to this study's. The two-POTS and three-POTS were presented side-by-side with the fixation stimulus. The POTS size, the task, and the experimental procedure were essentially the same as in Experiment 2. Eight participants showed that the mean disparity of the two-POTS with 4.49 dots/deg^2^ dot density producing the same apparent depth as the three-POTS with the 18.34 arcmin was 15.83 arcmin (95% CI, 14.54–17.12), 16.66 arcmin (95% CI, 15.11–18.21), or 16.74 arcmin (95% CI, 15.57–17.91) for the 2.25, 4.49, or 8.98 dots/deg^2^ condition, respectively. We observed the depth reduction phenomenon, and the dot density did not affect it.

The frame’s depth enhancement helps to infer the mechanisms mediating the phenomenon of depth reduction in the POTS. The idea proposed for stereo-transparency^26^ can explain the depth reduction phenomenon with some additional assumptions. The idea assumes that a process assigns the disparity of dots in the backmost surface of a POTS configuration to their surrounding “blank” areas to form an opaque surface and increases neural activities representing the back surface. Aida et al.^22^ argued that the neural activity increases the perceived depth of a two-POTS configuration and introducing another surface(s) weakens the bias. The argument is consistent with our results that the perceived depth of the two-POTS configuration was larger than those predicted from the geometry and getting closer to those as a function of the number of surfaces. Furthermore, the assumption that there is inhibitory interaction at the level of a surface representation in stereo transparency can explain the phenomenon^21,22^.

In stereoscopic processing, the mechanism responsible for the depth reduction of a POTS configuration may operate after the stereoscopic transparency is achieved. Aida et al.^22^ found that the cross-correlation analysis did not fit well with their finding and argued that the phenomenon might not be due to an early disparity detection process but a higher-order disparity detection process representing stereo surfaces. In the visual stream, the disparity is processed "sequentially" at several stages. The human function MRI studies using a two-POTS configuration suggest that the neurons in V3A and MT + played a role in processing the configuration^47,48^. The depth reduction phenomenon may occur after disparity processing at the cortical area.

To sum up, we confirmed that the perceived depth magnitude of a 3-D stereoscopic transparent stimulus consisting of multi-parallel overlapping stereoscopic surfaces decreases as a function of the number of surfaces and reported that the stimulus’ perceived depth magnitude increases when surrounded by a frame. The depth reduction phenomenon effect in stereoscopic stimuli with multiple surfaces indicates that the geometry does not simply determine the depth magnitude. The depth enhancement effect of the frames suggests a global process to combine the outputs of disparity processing of a stimulus surrounded and a frame surrounding. The global process concerning disparity processing has also been found in the frame’s depth enhancement effect of a 2-D photo and the literature on depth contrast.

## Methods

### Apparatus

The stimuli were generated using MATLAB and Psychotoolbox on a Windows PC (LAVIE Direct DT PC-GD289ZZDL, NEC, Tokyo, Japan). The stimuli were presented on the monitor (CS230-CN, EIZO, Hakusan, Japan) with a resolution of 1920 by 1080 pixels. Participants sat on a chair 40 cm from the floor in a dark room, placed their chins on a chin rest, and viewed the stimuli from 60 cm through a stereoscope. The experimental room was darkened, but the upper and lower edges of the monitor were visible when stimuli were presented. (Thus, the visible edges could affect the perceived depth magnitude. However, if any, the effect would likely be similar among present experimental conditions.)

### Stimuli

**Figure 4:**
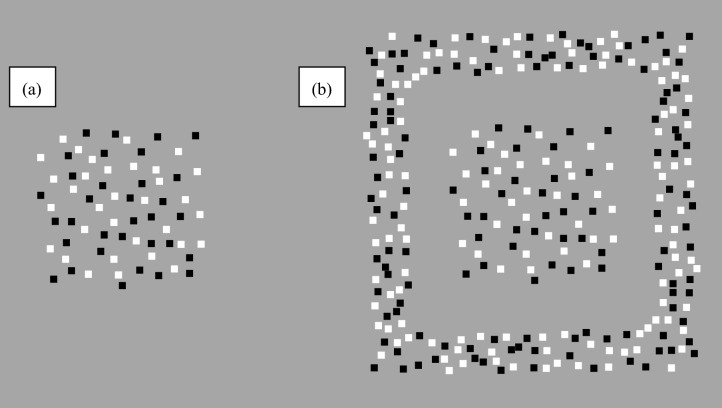
Schematic front view of a fused stimulus without a frame (**a**) and without it (**b**).

**Figure 5:**
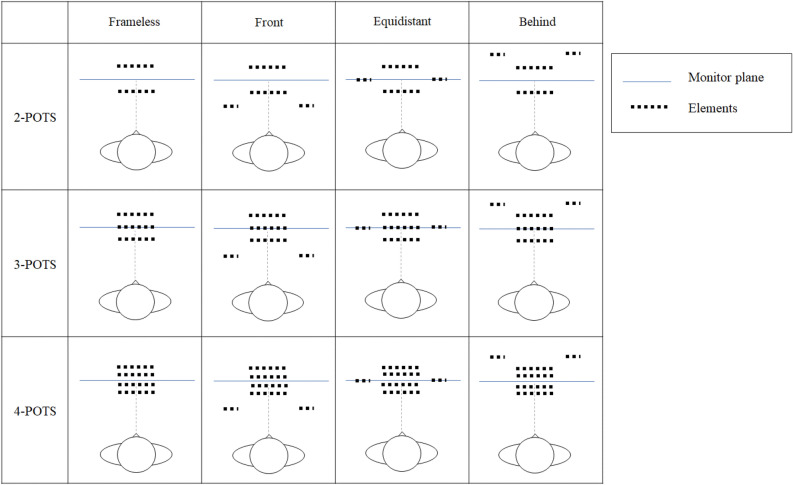
Schematic top view for fused stimuli. From top to bottom, the views were for two-, three-, and four-POTS configurations. From left to right, the views were the frameless, front-frame, equidistant-frame, and back-frame conditions. The blue line represents the monitor plane, and the black rectangular box represents the position of each dot. While we depicted the POTS configuration and the frame as comprised of regularly deployed black dots, they are a texture of a random-dots pattern. See the text for a detailed description.

The stimuli were RDSs of 50% white and 50% black square dots (0.10 × 0.10 arcdeg) on a gray background. The luminances of white and black dots were 19.49 and 0.23 cd/m^2^, respectively, and that of the background was 9.49 cd/m^2^. When fused, the stimuli appeared as multiple surfaces (two, three, or four) located at different depth planes from a participant but in the same visual direction. Figure 4a,b show a schematic front view of the fused stimulus with and without a frame, respectively. The frame appeared in front of or behind a POTS configuration or at the monitor plane. Figure 5 schematically illustrates the fused stimulus' top view for each of the three POTS and four frame conditions.

In Experiment 1, we presented the stimulus at the center of the display. The frameless POTS was 10.10 × 10.10 arcdeg in size. The two-, three-, and four-POTS contained 336 dots (3.29 dots/deg^2^). In the two-, three-, and four-POTS, each surface consisted of 168 dots (1.65 dots/deg^2^), 112 dots (1.10 dots/deg^2^), and 84 dots (0.82 dots/deg^2^), respectively. The total disparities (inter-surface disparities summed up) between its closest and farthest surfaces were 18.33, 27.50, and 36.67 arcmin. These magnitudes will be called small, medium, or large, respectively. The inter-surface disparities of the two-POTS were 8.94 and − 9.40 arcmin, 13.25 and − 14.28 arcmin, and 17.46 and − 19.29 arcmin; those of the three-POTS were 8.94, 0.00, and − 9.40 arcmin, 13.25, 0.00, and − 14.28 arcmin, and 17.46, 0.00, and − 19.29 arcmin; and those of the four-POTS were 8.94, 3.03, − 3.08, and − 9.40 arcmin, 13.25, 4.53, − 4,64, and − 14.28 arcmin, and 17.46, 6.01, − 6,21, and − 19.29 arcmin. Positive and negative values represented crossed and uncrossed disparities concerning the monitor. The frame’s outer and inner dimensions were 12.36 × 12.36 and 11.11 × 11.11 arcdeg, respectively, and contained 400 dots (13.60 dots/deg^2^). Concerning the monitor plane, the frame had three binocular disparities, − 24.44, 0.00, and 21.56 arcmin. Negative (uncrossed), zero, and positive (crossed) disparities indicate that the frame appeared behind, at the same depth, and in front of the monitor plane, respectively.

In Experiment 2, two-POTS (or three-POTS) with and without a frame were displayed on the display, with a horizontal offset of 2.79 arcdeg. A fixation cross (0.25 × 0.25 arcdeg, 0.05 arcdeg stroke width) was placed at the center between the two POTS stimuli. The POTS was 9.60 × 5.57 arcdeg in size and contained 240 dots (4.49 dots/deg^2^). The two- and three-POTS had 120 dots (2.25 dots/deg^2^) and 80 dots (1.50 dots/deg^2^) per surface, respectively. The frameless POTS was used as a reference and had the same total disparity as in Experiment 1 (18.34, 27.53, or 36.75 arcmin). The total disparity of the framed POTS varied from 6.11 to 30.60, 15.28 to 39.60, or 21.40 to 52.19 arcmin in five-step increments for the reference with the small, medium, and large disparities, respectively. The frame had outer and inner dimensions of 11.86 × 7.84 and 10.61 × 6.58 arcdeg, respectively, and contained 320 dots (13.79 dots/deg^2^). The frames had two binocular disparities (0.00 and 26.73 arcmin) concerning the monitor.

In Experiment 3, a two- or three-POTS with or without a frame was presented at the center of the display as in Experiment 1. The POTS was 7.59 × 7.59 arcdeg in size and contained 240 dots (4.17 dots/deg^2^). The two- and three-POTS had 120 dots (2.09 dots/deg^2^) and 80 dots (1.39 dots/deg^2^) per surface, respectively. The total disparity of the POTS was the same as used in Experiments 1 and 2. Three different-size frames were used: the outer and inner dimensions were 10.10 × 10.10 and 8.85 × 8.85 arcdeg, 11.36 × 11.36 and 10.10 × 10.10 arcdeg, and 12.61 × 12.61 and 11.36 × 11.36 arcdeg, respectively, for the small, medium, and large size frames. The frame contained 424 dots (17.79 dots/deg^2^), 480 dots (17.82 dots/deg^2^), and 536 dots (17.85 dots/deg^2^) for the small, medium, and large size frames, respectively. The frame had zero disparity concerning the monitor plane.

### Procedure

Experiments 1, 2, and 3 included practice and main trials. The practice trials were administered until participants demonstrated comprehension of the task. The stimuli for the practice trials were randomly selected from those in the main trial. Participants indicated the number of overlapping surfaces before each main trial in Experiments 1 and 3. In all the experiments, no time limit was imposed for stimulus observation based on our preliminary observations; when a POTS configuration was presented with short durations, some participants had difficulty perceiving stereo-transparent surfaces, as reported in the literature^21,22^, and judging the perceived depth magnitude confidently. In Experiments 1 and 3, the participants could move their eyes, and in Experiment 2, they were instructed to maintain fixation on the fixation cross on the monitor. We discussed the possible role of the eye position on the perceived depth measured in this study in the “Discussion” section. The participants were allowed to take breaks whenever their eyes felt fatigued.

In Experiment 1, we asked participants to report the surface number of the POTS configuration and accurately replicate the extent of depth between the outer surfaces of the configuration. Participants held a tape measure and reproduced the depth magnitude by alternatively looking at the stereoscope and the measure at hand. They completed four experimental sessions. Each session's frames, total disparities, and surface numbers were randomly selected from four different frames, three different total disparities, and three different surface numbers of POTS, respectively, with one repetition. Thus, each participant had 144 trials (4 frames × 3 total disparities × 3 surface numbers × 4 repetitions).

In Experiment 2, participants were required to (1) assess the perceived depth between the outermost surfaces for the right and left side stimuli on the screen and (2) indicate which exhibited a superior magnitude of depth. The stimuli were visible until participants finished providing their estimates. The experiment consisted of twelve blocks that differed in two disparities of the frame, three total disparities of the frameless POTS, and two surface numbers of POTS. Each block comprised five sessions. For each session, the total disparities of the framed two-POTS or three-POTS stimulus and their spatial positions (right or left) were randomly drawn from five disparities and two locations, respectively, with one repetition. Consequently, each participant completed a total of 600 trials (2 frames × 3 total disparities of a frameless POTS × 2 surface numbers × 5 disparities of a framed POTS × 2 position × 5 repetitions).

In Experiment 3, participants performed a depth reproduction task as in Experiment 1. They completed ten sessions, and the frames, total disparities, and surface numbers for each session were randomly selected from four different frame sizes, three different total disparities, and two different surface numbers of a POTS, with one repetition. Thus, each participant had a total of 240 trials (4 frame sizes × 3 total disparities × 2 surface numbers × 10 repetitions).

### Participants

Nine participants, including this paper's first and second authors, took part in Experiments 1, 2, and 3. Of these participants, 11 were male, and one was female, aged between 22 and 36. We used the stereo butterfly test (Stereo Optical Company, Inc., Chicago, USA) to assess the participants' stereopsis. We found that they all had stereopsis for disparities of no less than 1.00 arcmin. Except for the authors, participants remained oblivious to the precise objectives of the investigation. Participants, except for the authors, were not informed of the precise objectives of the investigation beforehand. We obtained informed consent from all participants before the experiments under the ethical guidelines outlined in the Declaration of Helsinki. The institutional review boards at Yamaguchi University approved the study's protocol.

### Institutional review board statement

The study was conducted in accordance with the Declaration of Helsinki, and approved by the Institutional Review Board of Yamaguchi University (protocol code 2021–022-01 and date of approval 31 August 2021; protocol code 2022–003-01 and date of approval 16 May 2022; protocol code 2023-003-01 and date of approval 27 April 2023).

### Informed consent statement

Informed consent was obtained from all participants involved in the study.

## Data Availability

The datasets used and/or analyzed during the current study available from the corresponding author on reasonable request.
